# Minimizing Risks in Anterior Endoscopic Cervical Discectomy Using Ultrasound-Guided Hydrodissection: A Technical Report

**DOI:** 10.7759/cureus.81309

**Published:** 2025-03-27

**Authors:** Chien-Hua Chen, Yu-Jen Lu, Meng-Ting Wu, Tsung-Ju Wu

**Affiliations:** 1 Neurosurgery, Clive Chen Clinic, Taichung, TWN; 2 Neurosurgery, Yuan Rung Hospital, Yuanlin, TWN; 3 Neurosurgery, Chang Gung Memorial Hospital, Taoyuan, TWN; 4 College of Medicine, Chang Gung University, Taoyuan, TWN; 5 Neurosurgery, Cheng-Hsin General Hospital, Taipei, TWN; 6 Regenerative Medicine, Reboot Clinics, Changhua, TWN; 7 Physical Medicine and Rehabilitation, Changhua Christian Hospital, Changhua, TWN

**Keywords:** cervical discectomy, disc, endoscope, hydrodissection, ultrasound

## Abstract

Anterior endoscopic cervical discectomy (AECD) is a minimally invasive alternative to anterior cervical discectomy and fusion (ACDF) for treating cervical disc herniation. Despite its advantages, AECD poses risks to delicate anatomical structures, including the esophagus, recurrent laryngeal nerve, inferior thyroid artery, and carotid artery. This report introduces a novel ultrasound-guided hydrodissection technique designed to enhance the safety of AECD by improving visualization and reducing iatrogenic injury. By employing ultrasound imaging, key structures can be identified in real time, while hydrodissection creates a protective space, minimizing tissue trauma when advancing the needle. This technique allows precise needle placement and facilitates a safer surgical approach. The integration of ultrasound guidance with the hydrodissection technique has the potential to reduce complications and improve procedural accuracy, making it a valuable adjunct to AECD.

## Introduction

Anterior endoscopic cervical discectomy (AECD) was developed following the success of lumbar endoscopic spinal procedures [1]. Prior to its introduction, anterior cervical discectomy and fusion (ACDF) was the standard treatment for cervical radiculopathy caused by cervical disc herniation (CDH). Long-term outcomes for the AECD and ACDF are comparable in terms of pain relief and functional improvement [2]. AECD offers advantages, including shorter operative times, reduced hospital stays, and faster recovery. The procedure is best suited for patients with soft CDH and preserved disc height. Complication rates were similar between these groups as well. The common complications are swallowing difficulty, recurrent disc herniation, hematoma, endplate collapse, and dysphonia [3]. However, ACDF has additional fusion-related complications, such as motion limitation, non-union, hardware failure [4], and adjacent segment disorders [5].

Standard AECD involves an anterior percutaneous approach under fluoroscopic guidance for selective discectomy and foraminal decompression. The procedure can be performed under general or local anesthesia. The patient is positioned supine on a radiolucent table with the neck extended. Fluoroscopic imaging and palpation help identify key anatomical landmarks, including the target disc level, carotid artery, and sternocleidomastoid muscle. The working zone between the carotid artery and trachea is identified by palpation, and an 18-gauge needle is introduced into the disc space under fluoroscopic guidance. The force of palpation should be strong enough to let the surgeon feel the tactility of cervical spinal columns and push carotid vessels laterally. During needle insertion, the surgeon need to maintain their force of compression by fingers to constantly open the working zone. Anterior-posterior (AP) and lateral views of fluoroscopy confirm the correct disc level and ensure an adequate needle trajectory, while intraoperative discography could stain the herniated disc fragment and identify the leakage status. The following steps include guidewire introduction, skin incision, dilation, working sheath placement, and fragments removal using an endoscope.

However, palpation alone does not allow visualization of blood vessels and nerves, increasing the risk of injury to the recurrent laryngeal nerve, inferior thyroid artery, esophagus, and carotid artery [6]. Ultrasound can provide real-time imaging of these critical structures, and ultrasound-guided hydrodissection could help clinicians advance the needle safely under guidance [7]. After adequate training, clinician could advance the needle without touching the tissue in front of the needle tip. Instead, injectate serves as the tool to separate the soft tissues in front of the needle, followed by advancement of the needle tip into the resultant fluid space. In this technical report, we integrate ultrasound and the ultrasound-guided hydrodissection technique to create a safe space between the carotid sheath and fascia visceralis, which covers the pharynx, larynx, thyroid gland, and esophagus [8]. Also, critical structures in the route of needle advancement like esophagus, carotid artery, thyroid, inferior thyroid artery, and recurrent laryngeal nerve could be identified, and needle-related trauma could be easily prevented by real-time monitoring of the procedure. This novel approach has the potential to enhance both the safety and precision of AECD.

The research was conducted in accordance with the principles outlined in the Declaration of Helsinki. Written informed consent was obtained from the patient for the publication of case details and any accompanying images. The institutional review board waived approval for this technical report, as it contains no identifiable information.

## Technical report

**Figure 1:**
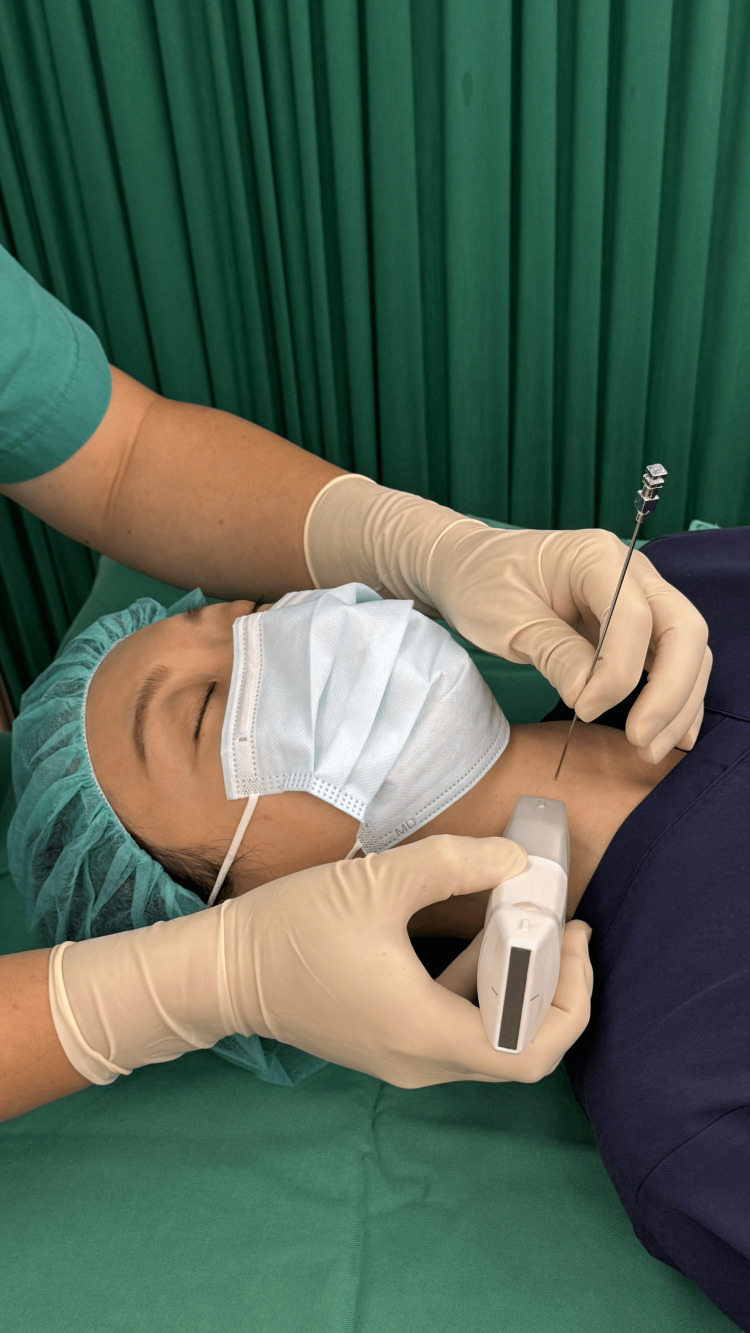
The patient is placed in the supine position on a radiolucent table with the neck extended. The skin overlying the working zone and the ultrasound probe are prepared using standard aseptic technique. As shown in the figure, the surgeon performs a medial-to-lateral approach after verifying the potential needle trajectory under ultrasound guidance.

For this technique, either a curvilinear or linear ultrasound probe can be used. The procedure may be conducted under either general or local anesthesia, based on the patient's condition and the surgeon's discretion. The patient is placed in the supine position with the neck extended on a radiolucent table. The skin overlying the working zone and ultrasound probe were prepared using standard aseptic technique (Figure 1).

**Figure 2:**
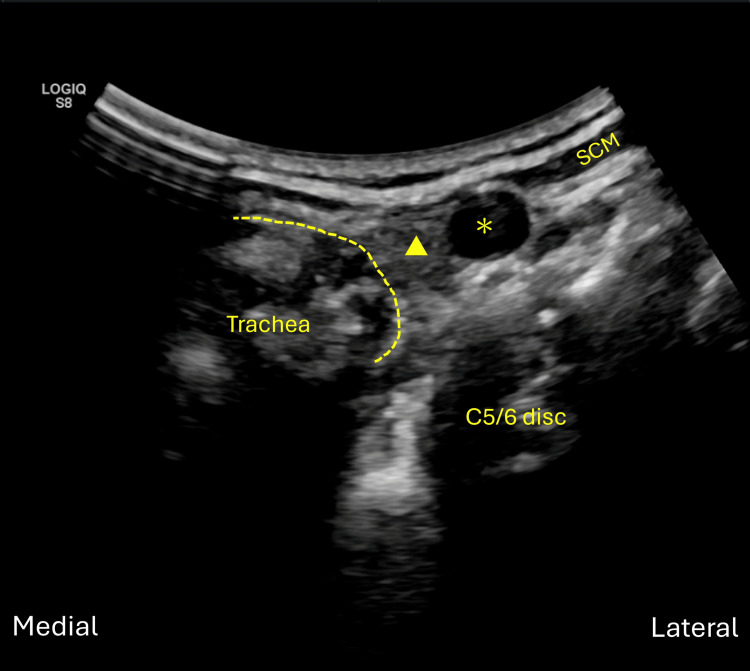
Identify the anterior tubercle of the C6 vertebra first in axial plane and sweep the probe in short axis cranially to C 5/6 disc. Fascia alaris (asterisk) and thyroid gland (arrowhead), which is part of fascia visceralis, could be seen above the cervical disc. The SCM muscle could also be observed laterally to the fascia alaris, and the trachea could be observed medially to the thyroid. SCM, sternocleidomastoid

The cervical level is determined by identifying the most prominent anterior tubercle of the C6 vertebra in the axial plane [9]. Also, the anterior tubercle of C7 is difficult to identify due to its small size, and these characteristics could also help localize the correct disc level. Counting from these bony landmarks allows precise localization of the target disc. In this article, we use the C 5/6 intervertebral disc as an example. Anterior tubercle of the C6 is identified first in axial plane, and we sweep our ultrasound probe in short axis cranially to C5/6 disc, which ultrasound beam can penetrate through it. Carotid sheath, also known as fascia alaris [8], and the fascia visceralis could be seen above the cervical disc. The sternocleidomastoid (SCM) muscle could also be observed laterally to the fascia alaris, and the trachea could be observed medially to the thyroid (Figure 2).

**Figure 3:**
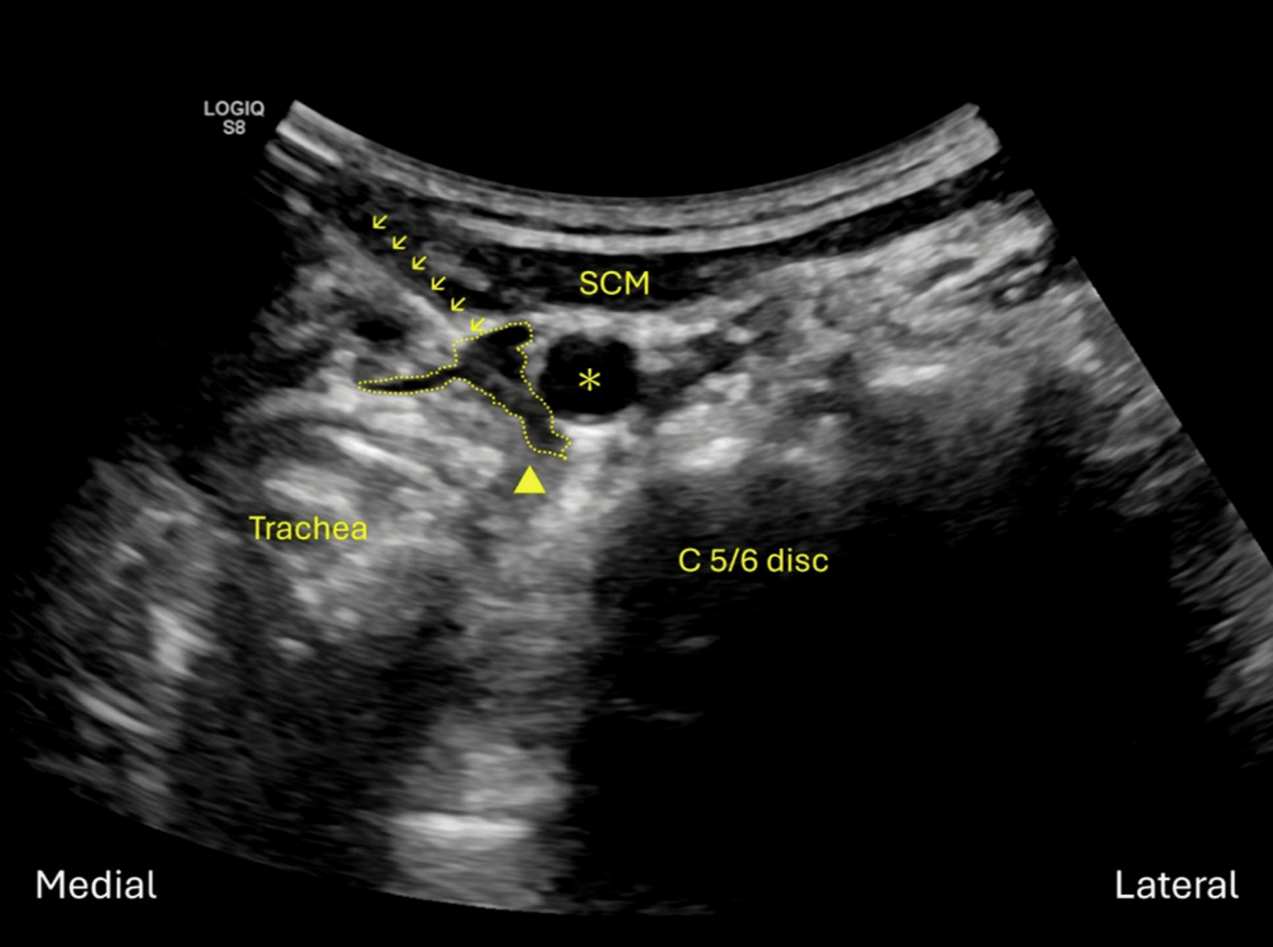
A needle is inserted with an in-plane approach medial to the SCM muscle. Then hydrodissection technique is performed to separate the space between the fascia alaris (asterisk) and the fascia visceralis (arrowhead). Now the open space is filled with hypoechoic fluid (dotted line), and the needle could advance without further injury to the fascia alaris and fascia visceralis. SCM, sternocleidomastoid

After locating the desired disc level and marking on the skin, a spinal needle without a stylet is connected to a syringe and inserted under ultrasound guidance. Power Doppler imaging (PDI) is used to identify small vessels in the route of needle advancement. The needle is introduced using an in-plane technique, aligned with the axial imaging plane of the cervical spine. After the needle is penetrating the skin and reaching the fascia layer medial to sternocleidomastoid muscle, hydrodissection technique is performed to separate the space between the fascia alaris and the fascia visceralis with fluid. The needle is then advanced to the space anterior to the longus colli or longus capitis muscle, depending on the target level (Figure 3).

**Figure 4:**
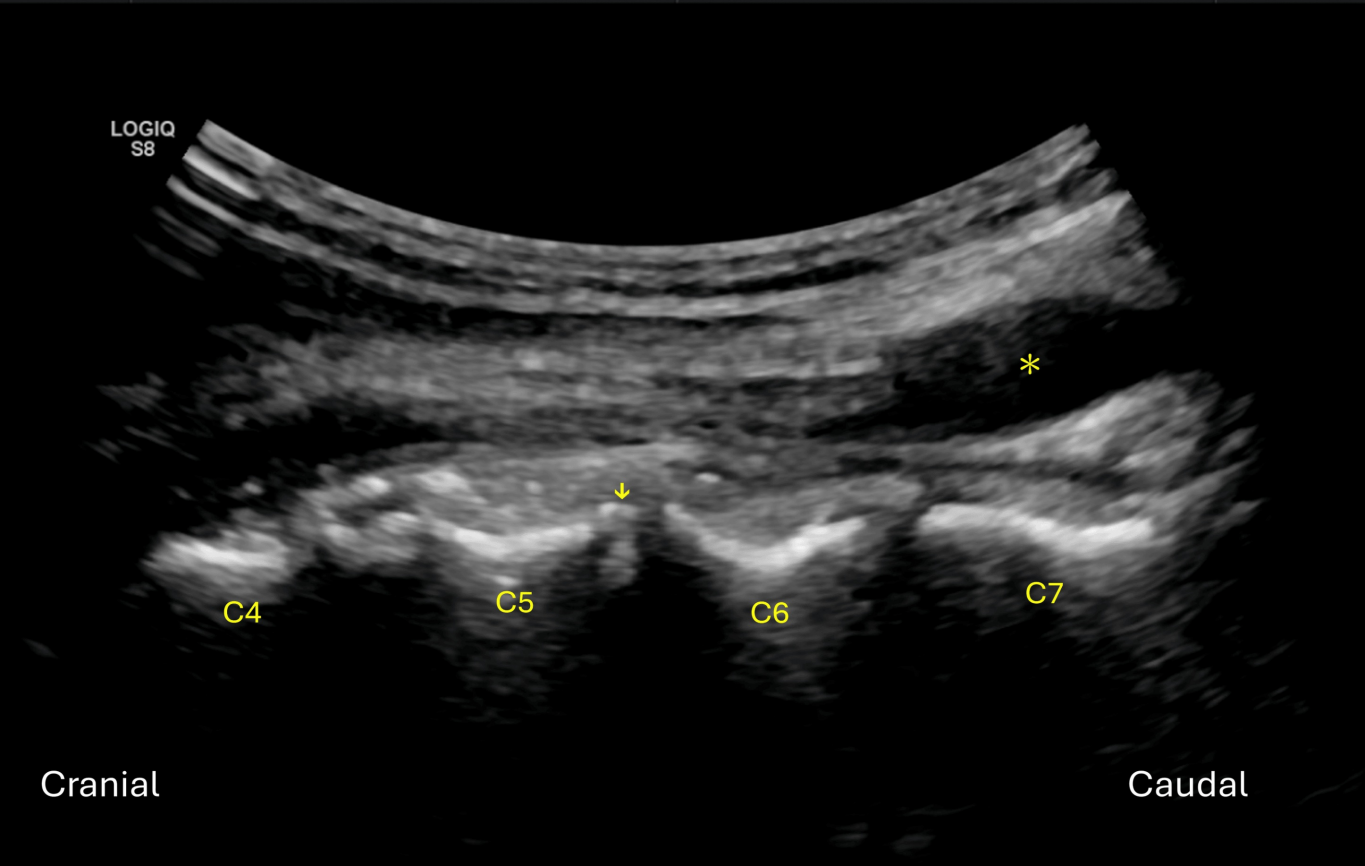
The probe is then rotated 90 degrees to the sagittal plane when the needle reaches the anterior surface of the disc. Fine-tuning of the needle tip position (arrow) to the anterior surface of the disc could be performed in the sagittal plane using the out-of-plane approach. Oblique axis of the common carotid artery (asterisk) could be seen as well.

The probe is then rotated 90 degrees to the sagittal plane to fine-tune the needle tip position to the anterior surface of the disc (Figure 4). The needle tip is inserted into disc using the out-of-plane approach. Ipsilateral or contralateral access could be chosen based on surgeons’ preference. Confirmation of the needle trajectory is achieved by switching back to the axial view. Verification of the disc level is achieved by AP and lateral fluoroscopy. Once the position is confirmed, a guidewire is inserted into the nucleus pulposus, allowing subsequent procedural steps to be performed safely.

## Discussion

AECD offers advantages such as shorter surgical time and quicker recovery compared to ACDF. However, complications such as esophageal injury, recurrent laryngeal nerve damage, and arterial injury can still occur. Complication rates between these groups are similar. Esophageal injury can lead to dysphagia, while hematomas and swelling may result from arterial injury. Damage to the recurrent laryngeal nerve can cause dysphonia. In the AECD procedure, the working zone between fascia alaris and fascia visceralis could be identified by palpation. The space is created by the consequent axial force of fingers until the surgeon feels the tactility of the anterior surface of the disc. When the needle is advanced from the skin to the anterior surface of the disc, the procedure is guided only by palpation. The angle of needle and level of desired disc level could only be verified by fluoroscopy, which is not a real-time monitoring. Furthermore, palpation and fluoroscopy do not allow visualization of small vessels and nerves, increasing the risk of injury to the recurrent laryngeal nerve, inferior thyroid artery, esophagus, and carotid artery.

Ultrasound serves as a tool to real-time monitor and visualize those critical structures and help surgeons plan their route of needle advancement. Due to the short distance from skin to intervertebral disc in the anterior neck and the absence of significant intervening bony structures, ultrasound imaging provides excellent clarity with minimal anatomical limitations. The esophagus, thyroid, and carotid artery can be easily identified on ultrasound. The recurrent laryngeal nerve, a branch of the vagus nerve, travels in the tracheoesophageal groove along the trachea and esophagus, passing posterior to the thyroid gland. It is a relatively small structure, typically located medial to the thyroid gland, and can be identified using high-resolution ultrasound imaging, along with the inferior thyroid artery [10]. Identification of the recurrent laryngeal nerve and inferior thyroid artery allows surgeons to plan a trajectory lateral to the thyroid gland, effectively minimizing the risk of nerve injury, as the nerve typically lies medial to the gland. Ultrasound can also help localize the desired disc level by identifying distinct anatomical features of the anterior cervical tubercles at different levels, thereby reducing radiation exposure for the patient and surgical team [9].

Beyond enhanced visualization, ultrasound-guided hydrodissection technique allows for safer needle placement and reduces the risk of iatrogenic complications. Clinicians can advance the needle without directly contacting tissues, gently separating tissue layers by fluids to create a protective buffer around the surgical corridor. Additionally, real-time ultrasound imaging allows surgeons to continuously visualize and adjust the needle trajectory during insertion. The integration of ultrasound and ultrasound-guided hydrodissection technique into AECD enhances precision and safety, making it a valuable adjunct for spine surgeons.

## Conclusions

The incorporation of ultrasound guidance into AECD, particularly with hydrodissection technique, has the potential to improve the safety of the procedure by identifying and preserving critical anatomical structures. We believe that this novel approach could reduce complications and enhance patient outcomes when compared with traditional fluoroscopic-guided percutaneous anterior cervical approach. Future larger studies or comparative trials are necessary to objectively assess complication rates, procedural efficiency, and long-term outcomes, and further validate the clinical advantages of ultrasound-guided hydrodissection in AECD.
